# Ectopic expression of a truncated CD40L protein from synthetic post-transcriptionally capped RNA in dendritic cells induces high levels of IL-12 secretion

**DOI:** 10.1186/1471-2199-9-90

**Published:** 2008-10-17

**Authors:** Irina Y Tcherepanova, Melissa D Adams, Xiaorong Feng, Atsushi Hinohara, Joe Horvatinovich, David Calderhead, Don Healey, Charles A Nicolette

**Affiliations:** 1 Research and Development Department, Argos Therapeutics Inc, Durham, NC, USA; 2 Becton Dickinson Diagnostic, Durham, NC, USA; 3 Kirin Pharma, LaJolla, CA, USA

## Abstract

**Background:**

RNA transfection into dendritic cells (DCs) is widely used to achieve antigen expression as well as to modify DC properties. CD40L is expressed by activated T cells and interacts with CD40 receptors expressed on the surface of the DCs leading to Th1 polarization. Previous studies demonstrated that ectopic CD40L expression via DNA transfection into DCs can activate the CD40 receptor signal transduction cascade. In contrast to previous reports, this study demonstrates that the same effect can be achieved when RNA encoding CD40L is electroporated into DCs as evidenced by secretion of IL-12. To achieve higher levels of IL-12 secretion, a systematic approach involving modification of coding and noncoding regions was implemented to optimize protein expression in the DCs for the purpose of increasing IL-12 secretion.

**Results:**

Site-directed mutagenesis of each of the first five in-frame methionine codons in the CD40L coding sequence demonstrated that DCs expressing a truncated CD40L protein initiated from the second methionine codon secreted the highest levels of IL-12. In addition, a post-transcriptional method of capping was utilized for final modification of the CD40L RNA. This method enzymatically creates a type I cap structure identical to that found in most eukaryotic mRNAs, in contrast to the type 0 cap incorporated using the conventional co-transcriptional capping reaction.

**Conclusion:**

The combination of knocking out the first initiation methionine and post-transcriptional capping of the CD40L RNA allowed for approximately a one log increase in IL-12 levels by the transfected DCs. We believe this is a first report describing improved protein expression of post-transcriptionally capped RNA in DCs. The post-transcriptional capping which allows generation of a type I cap may have broad utility for optimization of protein expression from RNA in DCs and other cell types.

## Background

RNA transfection into Dendritic Cells (DCs) is widely employed to achieve antigen expression [1]. RNA-transfected DCs are potent immune stimulators that have been tested in several clinical trials in cancer patients [2]. Transfection of DCs with RNA has several advantages over other platforms of antigen delivery such as DNA or viral vector-encoded antigens. Conditions may be optimized for cytoplasmic delivery of the RNA and together with transient expression and rapid degradation it may contribute to the safety of DC-based immunotherapy.

Successful protein expression from transfected RNA depends on transfection efficiency, translation competence, and stability of the transfected RNA. A 5' cap structure and 3' poly(A) tail are essential components for RNA translation in eukaryotic cells. Mockey *et. al*. demonstrated that a poly(A) tail of 100 nucleotides and a 5'ARCA cap analogue act synergistically to produce high protein expression in dendritic cells [3]. Holtkamp *et al*. reported that a longer poly(A) tail of 120 A residues as opposed to a more conventional poly(A) tail of 64 bases achieves higher protein expression levels. Furthermore, the 3' ends of the transfected RNA molecules were modified with a non-translated region from the β-globin gene of *X. laevis*. [4]. Together these modifications resulted in greater protein expression in DCs allowing for increased cell-surface presentation of epitopes and better induction of T cell responses.

In addition to antigen-MHC complexes the immunopotency of DCs is dependent on high levels of co-stimulatory molecules and secreted cytokines. Together, these elements conspire to induce antigen-specific T helper cell 1 (Th1) and cytotoxic T lymphocyte (CTL) responses. Transfection of DCs with RNA encoding for a pro-inflammatory cytokine can modify the DC phenotype to enable these desirable properties. For example, DCs co-transfected with tumor antigen RNA and IL-12 mRNA were shown to induce higher numbers of tumor-specific CTLs with greater functional avidity compared to those transfected with tumor antigen mRNA alone [5].

In the present study IL-12 secretion by DCs was achieved by a different approach involving transfection of the DCs with CD40L RNA. CD40L is normally expressed transiently on the surface of activated CD4+ T cells [6] and mediates interactions with cells such as DCs that express its receptor, CD40 [7]. The CD40L molecule interacts with CD40 expressed on immature DCs (iDCs) and other antigen presenting cells delivering a contact-dependent signal that drives DC maturation which enhances immunopotency [8]. CD40L is essential for the generation of CTL responses by RNA-transfected DCs [9]. Also, CD40L expressed in DCs from a Lentiviral construct induces phenotypic maturation as measured by up-regulation of the surface markers CD83, CD80, and MHC I, as well as secretion of IL-12 cytokine [10].

Here we demonstrate that expression of CD40L from transfected RNA likewise leads to increased maturation of DCs and induction of IL-12. Furthermore, additional optimization of the CD40L RNA was undertaken to achieve greater levels of the CD40L protein expression which in turn induce higher levels of IL-12 cytokine secretion. The first of these modifications was to the CD40L coding sequence where the initiation of translation from the second in-frame methionine codon led to greater levels of protein expression and consequently induced higher levels of IL-12 secretion. The other modifications we explored were not specific to the CD40L coding sequence and involved enzymatically adding a long poly(A) tail and post-transcriptional capping of the RNA.

In eukaryotic cells, nascent mRNAs are modified with a cap structure early during their transcription, when the transcript reaches 20–30 nucleotides in length [11]. First, the 5' terminal pppN of the RNA transcript is converted to 5' GpppN by a bi-functional capping enzyme containing both RNA 5'-triphosphatase and guanylyltransferase activities [12]. The GpppN moiety is subsequently methylated by a second enzyme containing (guanine-7)-methyltransferase activity to form the monomethylated m7GpppN Type 0 cap structure. The Type 0 cap is then converted to an m7GpppN Type 1 structure in the nucleus by 2'-O-methylation [13]. When RNA molecules are enzymatically synthesized *in vitro *using most commercially available kits, 5' capping occurs by a very different mechanism. Capping does not occur after synthesis of the transcript, but rather occurs concurrently with the initiation of transcription and is referred to here as "co-transcriptional capping". During the *in vitro *transcription reaction, the dinucleotide cap analog m7G(5')ppp(5')G (referred to from here on as m7G) is substituted for a portion of the GTP nucleotide in the reaction and transcription templates from which the RNA is transcribed are engineered such that the first nucleotide transcribed is a guanosine. Consequently, RNA transcription can be initiated with the cap analogue instead of GTP in a fraction of the molecules. A molar excess of cap analogue relative to GTP drives the reaction toward preferential incorporation of the cap dinucleotide at the first position of the transcript. The products of this type of transcription reaction are always mixtures of uncapped and capped RNAs resulting from initiation of synthesis with either GTP or the cap analogue, respectively. Uncapped molecules present in a synthetic mRNA preparation are typically not able to be translated upon transfection into eukaryotic cells (reviewed in [14]). In addition, the cap stabilizes the RNA since uncapped RNA is more susceptible to degradation by exonucleases in cells than the same RNA with an m7GpppN cap structure [15]. Therefore, the effective concentration of active molecules present in any preparation of co-transcriptionally capped RNA is less than 100% and is directly linked to the RNA capping efficiency achieved during the transcription reaction. The effective concentration of co-transcriptionally capped RNAs with the standard m7G cap analogue is further reduced because the cap analogue is able to be incorporated in the reverse (Gpppm7G) orientation up to 50% of the time [16]. Based upon the structural characterization of the interaction between the 5' mRNA cap and the protein synthesis machinery, it is expected that RNAs with a reverse cap orientation are not competent for translation [17,18]. Considering the issues of both capping efficiency and cap orientation, it is apparent that in a given preparation of co-transcriptionally capped RNA only a fraction of the RNA actually provides an active template for protein synthesis. The problem of cap analogue orientation in co-transcriptionally capped RNAs was addressed by Stepinski, *et al*. with the synthesis and characterization of the novel "anti-reverse" cap analog 7-methyl (3'-O-methyl) GpppG. This anti-reverse cap analog (ARCA) is incapable of being incorporated in the reverse orientation and thus gives rise to co-transcriptionally capped RNA preparations in which all capped transcripts should be competent for translation [19]. While most of literature on mRNA expression optimization utilizes ARCA [3-5], we attempted to further improve the translation competence of RNA by utilizing a method that poduces nearly 100% capped RNA. This process makes use of the Vaccinia Virus Capping Enzyme (VCE) which possesses all three enzymatic activities required to build an m7G-cap on the 5' end of synthetic mRNA molecules. These activities, RNA 5'-triphosphatase, guanylyltransferase, and guanine-7-methyltransferase, use GTP as a substrate [20-22] and are now commercially available reagents. The process by which the m7G cap is assembled on an uncapped synthetic RNA by the VCE is referred to here as "post-transcriptional capping" and results in RNA caps that are exclusively in the correct orientation. Post-transcriptional capping can also be used to assemble a 5' ARCA cap on RNA if 3'-O-Methyl GTP is substituted for the GTP precursor. In addition a type I cap may be created by adding a second Vaccinia enzyme, 2' O methyltransferase, to the capping reaction. RNA carrying type I caps are reported to have enhanced translation efficiency compared to those modified with the type 0 caps. Furthermore, most eukaryotic RNAs have type I caps. Thus, the ability to generate type I RNA is a significant improvement over traditional methods of RNA capping. To our knowledge this is the first report documenting expression of RNA modified with Type I cap in the DCs. Since this RNA modification in not gene sequence-specific, it may be broadly applicable for increasing protein expression from any synthetic RNA molecule.

## Results

### DCs electroporated with CD40L RNA induce IL-12 cytokine

**Figure 1:**
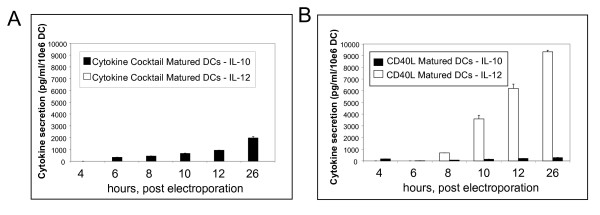
**DCs electroporated with CD40L RNA secrete IL-12**. Panel A: Cytokine secretion profile of DCs transfected with 2 μg/million DC eGFP RNA and matured with the cytokine cocktail. Panel B: time course for the secretion of IL-10 versus IL-12 for DCs matured by transfection with 4 μg/million DC CD40L RNA and cultured in IFN-γ/PGE_2_. Panel C: IL-10 and IL-12 release as well as expression of the DC maturation markers, CD80 and CD83 from immature DC transfected with a range CD40L RNA concentrations, and immediately matured in the presence of IFN-γ.

Since antigen loading of DCs is achieved via RNA transfection, the antigen payload can be conveniently supplemented with the CD40L-encoding RNA. Figure 1 shows that iDCs matured with the commonly used cytokine cocktail consisting of TNFα, IL1β, IL6 and PGE2 do not secrete IL-12, but instead they release low levels of IL-10. In contrast, IL-12 is detected in the supernatants of CD40L RNA-transfected cells from 8 hours post-transfection (Figure 1).

A dose response experiment between the mass of RNA electroporated into iDCs and the maturation phenotype was performed (Figure 1). The levels of expression of the maturation markers, CD80 and CD83, as well as the amount of secreted IL-12, directly correlated with the amount of CD40L RNA transfected (unpublished data).

These data support earlier observations that CD40L RNA expressed ectopically in DCs induce DC maturation and induction of IL-12 [10]. The level of IL-12 secretion varied from donor to donor. Examples of IL-12 variability observed using this method are summarized in Table 1. A wide range of IL-12 cytokine production as a function of donor to donor variation is well documented by others [23]. However, unlike the previous report, we achieve the effect from transiently expressed RNA rather than recombinant lentivirus transduction. Although the desired effect of DC maturation and Th1 polarization was achieved, further optimization of expression was performed.

**Table 1:** IL-12 cytokine levels in the supernatants of electroporated DCs

	IL-12, pg/mL		
cellular material used	CD40L WT	CD40L DXE	*Fold change
donor 1	326	8068	24.7
donor 2	31076	133712	4.3
donor 3	5717	35796	6.3
donor 4	2417	36572	15.1
donor 5	8085	58532	7.2
donor 6	7207	20954	2.9

*fold-increase in IL-12 detected by Elisa in DC supernatants 24 hrs post electroporation with CD40LDXE RNA compared to WT RNA

### Examination of the 5' Untranslated Sequence within CD40L RNA

**Figure 2:**
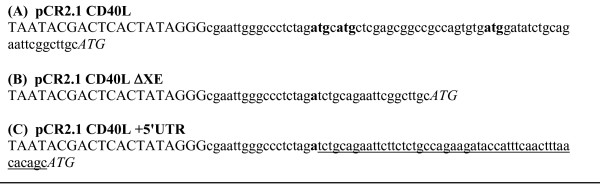
**Nucleotide sequence preceding CD40L initiator methionine in various CD40L RNA transcription templates**. Upper case: T7 promoter sequence; lower case: the sequence of 5'UTR; Upper case italics: accurate CD40L initiation codon; Bold lower case: upstream initiator codons which are not natural initiator codon for CD40L; Underline: the natural CD40L untranslated sequence (5'UTR).

CD40L mRNA stability is known to be regulated in T-cells through sequence elements within the 3' UTR [24]. During the original cloning of the CD40L transcription template, the coding region was PCR-amplified from an activated T-cell cDNA library and only minimal 5' and 3' UTR sequences were included. The majority of the UTR sequences flanking the transcribed CD40L coding region were therefore plasmid sequences. As a first step toward optimization of the CD40L RNA sequence, the 5'UTR region of the RNA was examined for elements that may be unfavourable with regard to mRNA stability or translation initiation. It was noted that the 5' UTR contained three potential ATG translation initiation codons upstream of the natural CD40L initiation codon (Figure 2), none of which are in the correct reading frame. It is known that such "upstream ATGs" can compete with the proper ATG codon for initiation of translation thereby reducing the rate and accuracy of translation initiation [25]. We postulated that such competition between potential translation initiation sites in the CD40L RNA may lead to suboptimal translation efficiency or inaccurate initiation in transfected DCs and account for the inconsistent expression of CD40L protein at levels sufficient for high IL-12 induction.

**Figure 3:**
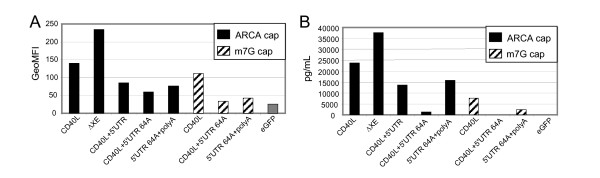
**CD40L protein expression and IL-12 cytokine secretion in DC cultures transfected with various CD40L RNAs**. Panel A: intracellular staining of DCs with anti-CD40L antibody at 4 hours post-electroporation with the RNAs. Panel B: cytokine levels measures in DC culture supernatants after overnight incubation. GFP is used as a negative control RNA. Type of cap analogue is indicated by shading back: ARCA and hatched m7G. All RNAs in this experiment were capped co-transcriptionally. All RNAs were polyadenylated in a post-transcriptional reaction and contained >150 nucleotide long poly(A) tail, except for CD40L+5'UTR 64A. For this RNA poly(A) tail was obtained in a transcription reaction by transcribing from templates containing poly(T) stretch after CD40L coding region. 5'UTR 64A+polA tail designates the RNA which in addition to 64 long poly(A) tail were post-transcriptionally polyadenylated. In this case poly(A) tail was >210 nucleotide long.

*In vitro *transcribed RNAs were prepared from each of the three plasmid templates listed in Figure 2. The RNAs were capped co-transcriptionally with either m7G or ARCA cap analogues. These RNAs contained either a 64A tail generated from *in vitro *transcription from a poly(T) containing DNA template or were polyadenylated after the transcription reaction in which the poly(A) tails were greater than 200 nucleotides. Four hours post-electroporation into DCs, CD40L protein expression was measured and secreted IL-12 cytokine levels were assayed after overnight incubation (Figure 3). Intracellular staining with anti-CD40L antibody indicates that the ARCA-capped CD40L ΔXE RNA is translated at the highest level relative to the other RNAs and also relative to the original CD40L RNA. For each distinct RNA sequence, comparison of ARCA- and m7G-capped RNAs demonstrates that ARCA-capped RNAs produce higher levels of CD40L protein. Additionally, CD40L+5'UTR RNA with a long poly(A) tail is translated to a higher level than the same RNA with a 64 nucleotide poly(A) tail. Interestingly, the results in Figure 3 (panel B) demonstrate that protein expression data at 4 hours post-transfection does not linearly correlate with the level of IL-12 secreted by the same cells at 24 hours post-transfection. In some cases, similar levels of CD40L protein expression induced dramatically different levels of secreted IL-12 (compare the +5'UTR RNA to +5'UTR+64A in Figure 3, panel B). Nevertheless, the ELISA results indicate that the ARCA-capped CD40L ΔXE RNA induces the highest level of IL-12 secretion, consistent with the protein expression data. The finding that the ΔXE RNA, which lacks the "upstream ATGs", is translated more efficiently and induces higher levels of cytokine secretion than CD40L RNA is in agreement with the idea that the "upstream ATG's" in the 5' UTR of the WT RNA interfere with its translation. The presence of the naturally occurring CD40L untranslated region in the CD40L+5'UTR RNA decreased the RNA's activity relative to the current wild-type RNA. Consistent with previous findings for the wild-type RNA, the +5'UTR RNA appears more active in inducing IL-12 expression when it contains an ARCA cap and a long poly(A) tail. However, since the naturally occurring CD40L 5' UTR did not elevate expression of CD40L RNA from the original construct this modification was no longer studied.

### Optimization of initiator codon and open reading frame

**Figure 4:**
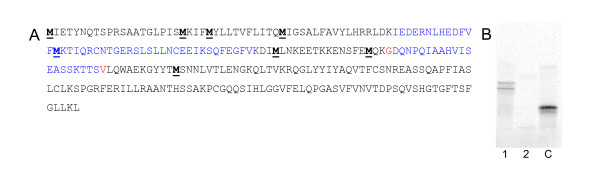
**Sequence analysis of coding CD40L protein**. Panel A: Amino acid sequence of the human CD40L full length polypeptide. In frame internal methionine residues are in bold and underlined. Panel B. SDS-Page Analysis of CD40L polypeptides translated *in vitro*. Lane 1: CD40L ΔXE; Lane 2: no RNA template control; Lane 3: control of irrelevant positive RNA translation.

Examination of the CD40L coding sequence revealed more in-frame ATG codons (in addition to the natural initiator ATG) within the first 108 nucleotides transcribed (Figure 4, panel A). *In vitro *translation products of CD40L ΔXE RNA reveal two polypeptide bands (Figure, 4 panel B). The upper band migrates with the expected mobility for full length protein whereas the lower molecular weight polypeptide may be a product of initiation at one of downstream in-frame Methionine codons.

**Figure 5:**
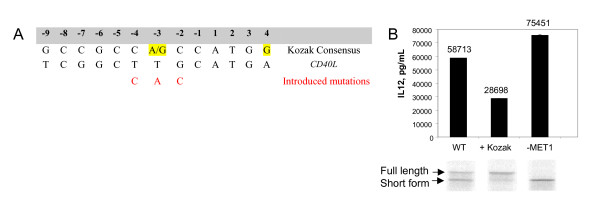
**Analysis of initiator methionine codon for Kozak composition and its influence on initiation of translation**. Panel A. Comparison between the eukaryotic Kozak consensus sequence and the sequence surrounding the CD40L translation initiation site. Nucleotides critical for efficient translation initiation are highlighted. Nucleotide substitutions predicted to enhance CD40L translation initiation without modifying the coding region are shown in red. Panel B. Correlation between CD40L small isoform expression and IL-12 induction. Graph demonstrates quantitative assessment of level of IL-12 cytokine secretion and below are shown SDS PAGE analysis of translation products. WT: wile type CD40L RNA, +Kozak: CD40L sequence with optimized first methionine codon; -Me1: CD40L sequence with knock out of first naturally occurring initiator methionine codon.

Closer inspection of the sequence surrounding the natural ATG codon for CD40L translation initiation revealed that the Kozak sequence surrounding the translation start site is not optimal. The Kozak sequence is a defined consensus sequence surrounding the ATG initiation codon in eukaryotic mRNAs that mediate efficient initiation of translation by ribosomes [26]. Figure 5 (Panel A) shows a comparison of the eukaryotic Kozak consensus sequence (initiator codon at positions +1 through +3) with the same 5' region in the CD40L sequence. The residue at position +4 cannot be modified without affecting the CD40L coding sequence, and therefore was not manipulated. The three nucleotide substitutions shown in Figure 5 (positions -4, -3, and -2) were engineered into the pCR2.1 CD40L ΔXE plasmid to create pCR2.1 CD40L ΔXE + Kozak. Site-directed mutagenesis was performed on the pCR2.1 CD40L ΔXE plasmid in order to change the first CD40L ATG codon to GCG, thereby generating the pCR2.1 CD40L ΔXE-MET1 plasmid. It was predicted that this mutation would result in translation initiation from an internal ATG codon within the ΔXE-MET1 RNA. It was also hypothesized that such internal initiation would result in predominant expression of the smaller of the two CD40L polypeptide species observed in the *in vitro *translation reaction (Figure 4, panel B). RNAs transcribed from these templates were assayed for the ability to induce IL-12 expression in the DC transfection assay (Figure 5 Panel B). In addition, the RNA was translated *in vitro *in the presence of ^35^S-labeled methionine and analyzed by SDS-PAGE and autoradiography.

We altered the sequence immediately upstream of the translation initiation site such that it more closely matches the optimal Kozak consensus sequence. As predicted, data presented in Figure 5 shows that this modification results in the synthesis of predominantly full-length protein. Surprisingly, this modification does not render the ΔXE + Kozak RNA more effective for induction of IL-12 expression compared to the CD40L RNA. CD40L protein expression measured at 4 hours post-electroporation displayed a similar trend (Data not shown). Interestingly, the CD40L-Met 1 RNA produced a single popypeptide migrating with the apparent mobility of the smaller polypeptide obtained in *in vitro *translation of a full length CD40L RNA. This RNA induced higher levels of IL-12 than CD40L RNA (Figure 5 panel B).

The lower relative molecular weight of this smaller, potentially more active polypeptide is consistent with its translation being initiated at one of the internal, in-frame methionine residues within the 5' end of the coding sequence. However, from this experiment the particular methionine residue at which protein synthesis initiates could not be delineated. Therefore, each of the next three CD40L methionine codons were mutated consecutively in the pCR2.1 CD40L-MET1 background to create the following templates: pCR2.1 CD40L-MET1,2; pCR2.1 CD40L-MET1,2,3; and pCR2.1 CD40L-MET1–4. ARCA-capped, polyadenylated RNAs were prepared from each of these transcription templates for analysis by *in vitro *translation and the DC transfection assay.

**Figure 6:**
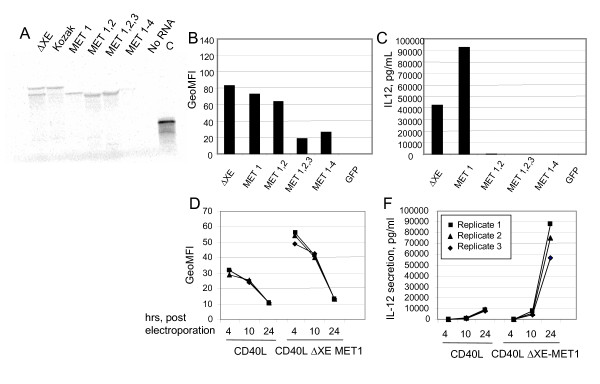
**CD40L protein lacking first naturally occurring Methionine codon induces highest levels of IL-12 secretion in DCs**. Panel A: SDS-Page Analysis of CD40L polypeptides translated *in vitro *obtained from RNAs with consecutively knocked out 1–4 methionine codons. No RNA: *in vitro *translation products of reaction containing no RNA template. C: translation of a positive control unrelated RNA Panel D: intracellular staining of DCs with anti-CD40L antibody of DCs harvested at indicated time point post-electroporation with either CD40L WT or CD40L ΔXE Met 1 RNAs as indicated on the bottom of the graph. Panel F: levels of IL-12 cytokine measured by ELISA accumulated in supernatants collected at indicated time point post-electroporation in DC cultures electroporated with either CD40L WT or CD40L ΔXE Met 1 RNAs. Each electroporation was performed in triplicate. All CD40L RNAs were ARCA-capped with a long poly(A) tail. GFP is a negative control RNA.

*In vitro *translation of RNAs transcribed from all templates in the presence of ^35^S-methionine demonstrated that only the ΔXE-MET1 RNA encodes a polypeptide species with a relative mobility equal to that of the smaller CD40L isoform (Figure 6 Panel A). The CD40L ΔXE-MET1,2 and ΔXE-MET1,2,3 RNAs both encode polypeptides of even lower molecular weight consistent with translation initiation at the fourth ATG codon. The CD40L ΔXE-MET1–4 RNA was not translated efficiently *in vitro*. These observations suggest that in vitro translation of the small CD40L isoform is initiated at the second ATG codon within the CD40L coding sequence. In transfected DCs, only the CD40L ΔXE-MET1, and ΔXE-MET1,2 were translated into detectable amounts of CD40L protein and the level of protein expression from the next RNA, ΔXE-MET1,2,3, was greatly reduced (Figure 6, panel B). This difference between the *in vitro *and *in vivo *expression data may suggest that the monoclonal antibody used for protein detection in DCs recognizes an amino-terminal epitope that overlaps the mutated region of the protein encoded by CD40L ΔXE-MET1,2,3. Alternatively, the epitope may become unavailable for antibody binding as a result of the -MET1,2,3 mutation. It is also possible that the ability of the RNAs to be translated in Wheat Germ Extracts does not accurately reflect the ability of the RNAs to be translated in DCs.

Although the ΔXE-MET1,2 RNA is capable of being translated into detectable protein in DCs, this RNA was not able to induce IL-12 secretion (Figure 6 panel C).

Next, a time course of protein expression and cytokine secretion was examined in DCs transfected with the two RNAs which induced the highest levels of secreted IL-12 (*i.e*., CD40L WT and CD40L ΔXE-MET1). The protein expression level in cells transfected with ΔXE-MET1 RNA is approximately two-fold greater at both 4 and 10 hrs post-electroporation (Figure 6 panel D). Both conditions resulted in almost undetectable intracellular CD40L expression at 24 hrs post-electroporation. The difference in duration of CD40L protein expression in the DCs showed even greater differences in induced IL-12 secretion. The total amount of the IL-12 cytokine secreted in 24 hours is almost a log greater in cultures transfected with the optimized RNA compared to wild-type RNA. This result is representative of several experiments performed on independently prepared DCs cultures from different donors.

### Post-transcriptional capping modification produces 100% capped, highly translation competent RNA

Data presented above is consistent with this expectation that induction of IL-12 secretion by DCs transfected with CD40L ΔXE MET1 RNA is more robust when the RNA contains an ARCA cap structure instead of an m7G cap due to the inability of ARCA to be incorporated into the inactive reverse orientation. Use of the ARCA cap analogue during CD40L ΔXE MET1 RNA production would address the problem of non-functional, reverse incorporation of the 5' cap. However, the final RNA product would still contain a mixture of capped and uncapped RNA molecules. To test whether IL-12 induction can be elevated even further we attempted to generate an RNA population with near 100% capping efficiency and therefore, 100% translationally competent.

**Figure 7:**
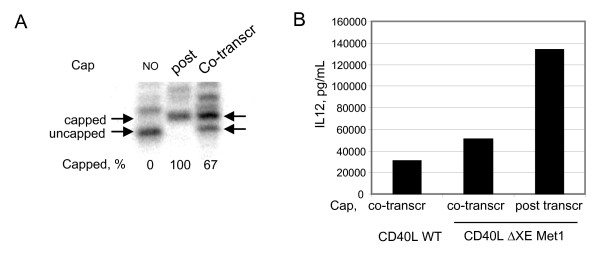
**Post-transcriptionally capped CD40L RNAs are 100% capped and induce the highest level of IL-12 secretion**. Panel A. Oligonucleotide-directed RNaseH cleavage products from the CD40L MET1 capping efficiency assay analyzed by denaturing polyacrylamide gel electrophoresis and autoradiography. Cleavage products from uncapped and capped RNA molecules are denoted by arrows. The control RNA is co-transcriptional ARCA-capped ΔXE-MET1 RNA. Capping efficiency measurements are indicated at the bottom of the gel image. Panel B: IL-12 cytokine secretion induced by ΔXE-MET1 RNA transfection as measured by ELISA. The resulting cap of each RNA is indicated by colour of the bars and as indicated, the type of cap (I or 0) is indicated below the graph. CD40L WT Reference RNA prepared with an ARCA cap by co-transcriptional cap analogue incorporation. GFP is a negative control RNA. All RNAs were polyadenylated post-transcriptionally and have >150 nucleotide tails. non-standard format).

The uncapped RNA was *in vitro*-transcribed from CD40L ΔXE-MET1 template and Type 1 m7G-capped RNA was generated. To measure the percent of capped RNA in a total population an oligonucleotide-directed RNAseH cleavage was performed. In this assay, the oligo anneals in the proximity to the 5' end of the RNA such that the size of the digested products are 19 nucleotides long if the RNA was not capped and 20 nucleotides long if the RNA was capped. The digested products can be radio-labelled and visualized by PAGE analysis. The capping efficiency measurements (Figure 7, Panel A) demonstrate quantitative RNA capping by the Vaccinia virus capping enzyme. This level of capping efficiency (100%) is never observed following traditional co-transcriptional cap analogue incorporation. Typically, co-transcriptional capping reactions results in RNA populations which are 60–70% capped when optimal conditions are used. In this experiment a quantitative measure of capping efficiency of co-transcriptionally capped RNA was 67% which is within the expected range for this type of reaction. The upper band in the uncapped RNA lane migrating above the band 21 nucleotide band is most likely a result of "RNA- oligo hybrid breathing" or altered conformation perhaps due to the absence of a cap structure. All of the RNAs were compared to one another and also to the original CD40L RNA in the IL-12 secretion assay (Figure 7 Panel B).

Consistent with previous observations the post-transcriptionally capped CD40L ΔXE-MET1 RNA was more potent for the induction of IL-12 secretion than the original construct. This observation was confirmed in several assays utilizing unique donor cellular materials (Table 1).

A comparative analysis of the post-transcriptionally capped RNAs indicates that the post-transcriptionally capped RNA has greater potency than either co-transcriptionally capped counterpart. The molecular precursor for the cap in the post-transcriptional reaction was GTP and the resulting structure closely resembles the m7G cap. However, 100% of the products of the post-transcriptional reaction using Vaccinia virus Capping enzyme are in the correct orientation compared to 67% of ARCA capped transcripts obtained in a co-transcriptional reaction. Since both types of caps are in a translation-competent form, the highest translation competency is observed in a population with greatest capping efficiency (*i.e*., post-transcriptionally capped ΔXE-MET1 RNA).

## Discussion

Untranslated sequences flanking an mRNA coding region are known to contribute to the post-transcriptional regulation of the RNA in eukaryotic cells, particularly with regard to mRNA stability and translation. As a first step in the improvement of translation efficiency of CD40L wild-type RNA it was postulated that the presence of cryptic ATG codons upstream of the correct ATG codon may create competition between potential translation initiation sites in the CD40L RNA that could lead to poor protein expression or inaccurate translation initiation in transfected DCs. This was confirmed by *in vitro *translation of the RNA which produced protein products of different molecular weights (Figure 4 panel B). This phenomenon may contribute to the inconsistent expression of CD40L protein levels required for high IL-12 induction. We observed that removal of the cryptic ATG codons alone increased the protein translation efficiency (Figure 5 panel B). For each distinct RNA sequence, comparison of ARCA- and m7G-capped RNAs demonstrated that ARCA-capped RNAs produce higher levels of CD40L protein. This observation is consistent with previous reports [3,5,19]. Furthermore, optimization of the Kozak sequence around the naturally occurring ATG codon led to initiation of translation exclusively from the first methionine. Surprisingly, this led to decreased levels of IL-12 by transfected DCs compared to the DCs transfected with CD40L RNA with an unoptimized Kozak sequence (Figure 5 panel B). In contrast, the RNA generated from the template lacking the first methionine produced higher protein levels and resulted in greater levels of IL-12 (Figure 5 panel B). The apparent correlation between predominance of the lower molecular weight protein isoform and the ability to induce high levels of IL-12 suggests that the small CD40L isoform is the more active signalling molecule when ectopically expressed in DCs.

Biological activity of a truncated recombinant 18 kDa fragment of CD40L protein was demonstrated previously [27]. Likewise the appearance of a shorter isoform was attributed to utilization of one of the internal in-frame methionines present in its sequence. The shorter protein isoform was also more biologically active, however the authors believed that the difference in activity was attributable to the more soluble nature of a shorter isoform versus the full-length protein. In our study, elimination of the first methionine by site-directed mutagenesis led to a deletion of the first 20 amino acids which does not predict disruption of the transmembrane domain by the Kyte and Doolittle hydropathy algorithm [28]. Another difference between these two studies is the use of extracellularly added recombinant form to the cell culture versus RNA which is ectopically expressed inside the cell.

The majority of RNA made in *in vitro *transcription reactions described so far utilize a co-transcriptional method of RNA capping. The ratio of capped to uncapped RNA produced can be modified by adjusting the ratio of cap analogue to GTP present in the reaction. Increasing the relative concentration of cap analogue enhances capping efficiency, however, the concomitant decrease in the level of GTP results in a lower yield of full-length product. The use of the post-transcriptional capping method that we employed results in a quantitative capping efficiency of 100% with all cap structures in the correct orientation. Thus, each molecule in this RNA preparation is translationally-competent. The m7G Type 1 cap obtained in a post-transcriptional capping reaction is indistinguishable from the cap structure found at the 5' end of naturally occurring mRNA's in eukaryotic cells and translation from RNAs modified with this cap structure is significantly enhanced compared to RNAs that do not contain the methylated ribose (*i.e*. type 0) cap [29].

In addition to the synergistic effect on higher protein expression, both the cap structure and poly(A) tail also contribute to greater stability of the RNA [3]. The poly(A) tail protects against initial degradation by exonucleases which is followed by de-capping [30,31]. The notion that greater poly(A) tail length can result in higher protein expression is well established [32]. It would be of interest to explore whether the difference in protein expression observed from identical RNAs generated with different cap structures is a consequence of the fact that one cap structure may be more resistant to de-capping enzymes.

Interestingly, the absolute level of protein expression measured at 4 hours post-transfection did not definitively predict the level of IL-12 secreted by the same cells at 24 hours post-transfection. It is possible that the induction of the IL-12 signalling pathway occurs at a time point later than 4 hrs post-electroporation and we are achieving greater steady-state levels as a result of increased RNA stability. Our routine Elisa assays studied IL-12 cytokine secretion at 24 hours post-electroporation. Human clinical studies examining intradermally administered ^111^indium-labeled DC migration show accumulation in the lymph node after just 6 hours and continued accumulation at 24 hrs and even 48 hrs post-injection [33,34]. It would be of interest to understand whether continued IL-12 secretion beyond 24 hours correlates with better clinical outcome.

Finally, another advantage of the post-transcriptional method is the ability to produce large amounts of uncapped RNA precursor using *in vitro *transcription reactions wherein the GTP nucleotide is not limiting. Transcription reactions that employ co-transcriptional RNA capping routinely include one-fourth the concentration of GTP that a standard reaction contains and consequently the final RNA yield from these reactions is approximately four times less than the theoretical maximum. In the process described here for CD40L MET1 RNA transcription, a four- to five- fold increase in yield from the same DNA template was obtained.

## Conclusion

In summary we demonstrate various RNA modifications that greatly improve the expression of CD40L protein from *in vitro *transcribed RNA and which may be applied to other RNAs for the purpose of enhancing protein expression.

## Methods

### Cloning of CD40L

Normal volunteer's T cells were stimulated with PMA for 1 hr. After stimulation and wash cells were used for RNA extraction. The RNA was taken into one tube RT-PCR reaction using Gene Amp Gold kit (Applied Bioscience) substituting Gene Amp Gold Taq polymerase with Advantage Polymerase (Clontech). One specific band of expected size of 0.8 kb was obtained in the PCR reaction using the following CD40L sequence specific primers: CD40L forward: 5'-GCATGATCGAAACATACAACC-3' and CD40L reverse : 5'-GTATTATGAAGACTCCCAGCG-3'.

Purified PCR fragment was subcloned into pCR2.1 vector. Presence of specific sequence was verified by restriction digest and sequencing. Results of the BLAST analysis performed on the final sequence indicated identity to sequence with accession # NM_000074 which is encoding for CD40L. One exception was A to G transition at a codon 102 of open reading frame which is silent and does not result in a change in amino acid sequence and was left unchanged.

### Modification of 5' end of CD40L template DeltaXE construct and 5' UTR of CD40L

To construct the pCR2.1 CD40L ΔXE plasmid, the pCR2.1 CD40L plasmid was digested simultaneously with XbaI and EcoRV (Promega). The XbaI overhang was subsequently filled with Klenow fragment of *E. coli *DNA Polymerase I (IDT) in the presence of 30 μM dNTPs. Following gel purification of the 4738 base pair DNA fragment was re-ligated using the FastLink DNA ligation kit (Epicentre Biotechnologies).

To construct the pCR2.1 CD40L+5'UTR plasmid, the pCR2.1 CD40L plasmid was used as a template in a PCR reaction with the following oligonucleotide primers: CD40L 5'UTR Forward (5'ACGAATTCTTCTCTGCCAGAAGATACCATTTCAACTTTAACACAGCATGATCGAAACATACAACC) and CD40L 3'UTR Reverse (5'CCAGTGTGCTGGAATTCGGC) and Pfu Ultra Hot Start (Stratagene). The resulting 890 bp PCR was then digested with EcoRI (Promega), gel purified and was ligated to EcoRI-digested pCR2.1 CD40L ΔXE plasmid. These manipulations resulted in the addition of 39 bp of human CD40L 5' UTR sequence 12 base pairs downstream of the transcription initiation site.

Construction of the pCR2.1 CD40L+Kozak plasmid involved PCR amplification of the pCR2.1 CD40L ΔXE plasmid template with the primers CD40L Kozak F (5'TCTGCAGAATTCGGCCACCATGATCGAAACATA) and CD40L 3'UTR Rev using Pfu Ultra Hot Start. The 880 base pair PCR product was digested with EcoRI and ligated to EcoRI site of pCR2.1 CD40L ΔXE.

The accurate translation initiation site in pCR2.1 CD40L ΔXE was mutated to create pCR2.1 CD40L ΔXE-MET1 using the QuickChange method described by Stratagene. The oligonucleotides used for mutagenesis were CD40L First Methionine F (5'CTGCAGAATTCGGCTTGCGCGATCGAAACATACAACC) and CD40L First methionine R (5'GGTTGTATGTTTCGATCGCGCAAGCCGAATTCTGCAG). This manipulation resulted in substitution of GC for AT in the CD40L first ATG codon. The changes were confirmed by DNA sequencing.

All subsequent methionine mutagenesis experiments followed the same procedure and resulted in a similar GC/AT substitution. The template for the pCR2.1 CD40L ΔXE-MET1,2 plasmid was pCR2.1 CD40L ΔXE-MET1 and the oligonucleotides used were CD40L Second methionine F (5'CACTGGACTGCCCATCAGCGCGAAAATTTTTATGTATTTACTTACTG) and CD40L Second methionine R (5'CAGTAAGTAAATACATAAAAATTTTCGCGCTGATGGGCAGTCCAGTG). The template for pCR2.1 CD40L ΔXE-MET1,2,3 was pCR2.1 CD40L ΔXE-MET1 and the oligonucleotides used were CD40L 2^nd ^& 3^rd ^methionine F (5'CACTGGACTGCCCATCAGCGCGAAAATTTTTGCGTATTTACTTACTG) and CD40L 2^nd ^& 3^rd ^methionine R (5'CAGTAAGTAAATACGCAAAAATTTTCGCGCTGATGGGCAGTCCAGTG). The template for pCR2.1 CD40L ΔXE-MET1–4 was pCR2.1 CD40L ΔXE-MET1,2,3 and the oligonucleotides used were CD40L Fourth methionine F (5'CTGTTTTTCTTATCACCCAGGCGATTGGGTCAGCACTTTTTGC) and CD40L Fourth methionine R (5'GCAAAAAGTGCTGACCCAATCGCCTGGGTGATAAGAAAAACAG).

### *In vitro *transcription of CD40L RNA

Linear templates for *in vitro *transcription were prepared by digesting each plasmid with SpeI (Roche). The linear template was purified by phenol:chloroform extraction followed by precipitation in ethanol and then resuspended in water.

Transcription reactions of co-transcriptionally capped RNAs were performed using mMessage mMachine T7 Ultra (Ambion). Transcription reactions to produce uncapped RNA were performed using T7 Flash Kit (Epicentre). Reactions were assembled as specified by each kit's manufacturer. All *in vitro *transcribed RNAs were purified using Qiagen RNeasy Purification kits as directed by the manufacturer.

### Post-transcriptional capping of CD40L RNA

Post-transcriptional capping modification of uncapped with type I cap CD40L MET1 RNA was performed using ScriptCap Kit (Epicentre Biotechnologies) components using capping buffer, GTP, SAM, Vaccinia Capping and Vaccinia 2'O-Methyltransferase Enzymes.

### Enzymatic polyadenylation of CD40L RNA

The polyadenylation was performed on purified RNA using A-plus Poly(A) Tailing Kits (Epicentre Biotechnologies). Final RNAs were purified as described above and the length of the poly(A) tail was determined by comparing sizes of pre- polyadenylated and polyadenylated RNAs on denaturing gel electropheresis. A typical preparation of post-transcriptionally polyadenylated RNA contained polyA tail of greater than 150 nucleotides.

### *In vitro *translation of CD40L RNA in the presence of ^35^S-Methionine

1 μg of RNA was heat denatured at 65°C for 3 minutes and immediately cooled on ice. 25 uL reaction containing the RNA and 1. 25 μL of Redivue ^35^S-labeled methionine (Amersham) was assembled using Wheat Germ Extract Kit (Promega) according to manufacturer's instruction. 1 μL of each reaction was resolved on SDS Denaturing gel electrophoresis and transferred onto PVDF membrane. The membrane was exposed to phosphoimager screen and image obtained by scanning using Storm imager (Amersham).

### Oligonucleotide-directed RNaseH cleavage products

2 μg of CD40L RNA and 32 pmol of the DNA oligonucleotide 5'CGCTCGAGCATGCAT/3ddC/3' (IDT) were first denatured at 85°C for 4 minutes and then allowed to hybridize for 4 hours at 42°C in 10 μL of RPA Hybridization III Buffer (Ambion). Immediately after the hybridization the RNA:DNA duplex was digested with 10 U *E. coli *RNaseH (Ambion) for 1 hour at 25°C. The products of the digestion were extracted with phenol:chloroform:isoamyl alcohol (25:24:1) and precipitated with sodium acetate and ethanol in the presence of 20 μg glycogen (Roche). The precipitate was washed with 70% ethanol, allowed to air dry and then resuspended in water. 3'end labeling was performed with 10 mCi/mL α^32^P-pCp (NEN Perkin Elmer), T4 RNA ligase buffer and 1.25U T4 RNA ligase (Ambion) for 12 -16 hours at 4°C. Labelled RNA was purified using NucAway spin column (Ambion). RNA samples were diluted with urea loading dye and resolved on an 8M urea/15% polyacrylamide gel. Reaction products were visualized in the wet gel by phosphorimager analysis and quantified using ImageQuant software.

### Generation of Immature DCs

Human PBMCs were isolated from Leukapheresis collections from healthy volunteers by Ficoll-histopaque density centrifugation. PBMCs were re-suspended in AIM-V medium (Invitrogen) and allowed to adhere to 150 cm^3 ^plastic flasks for 2 hours at 37°C. Non-adherent cells were removed and remaining cells cultured in X-vivo 15 medium, supplemented with 1000 U/ml GM-CSF (Leukine) and 1000 U/ml IL-4 (R&D systems), for 6 days at 37°C, 5% CO_2_.

### Generation of cytokine cocktail matured DC

Prior to electroporation, DCs were harvested and washed in PBS and then re-suspended in chilled Viaspan. DCs were mixed with 2 μg of mRNA encoding GFP per million cells.and placed in a 4 mm gap electroporation cuvette and electroporated using BioRad apparatus. Immediately after electroporation, DCs were washed in X-vivo-15 medium and finally re-suspended in X-vivo-15 supplemented with 800 U/mL GM-CSF and 500 U/mL IL-4 at 1 × 10^6^/ml. Maturation was achieved by addition of 10 ng/ml TNF-α, 10 ng/ml Il-1β, 100 ng/ml IL-6 and 1 μg/ml PGE_2 _followed by culture for 24 hours at 37°C in low adherence six well plates (BD Biosciences).

### Generation of CD40L matured DCs

As described above, washed immature DC were re-suspended in chilled Viaspan and mixed with varying amounts of mRNAs encoding CD40L as described in Results, and electroporated using BioRad apparatus. Post-electroporation, DC were cultured in X-vivo-15 in the presence of GM-CSF (800 U/ml) and IL-4 (500 U/ml) plus 1000 U/mL IFN-γ and 1 μg/mL PGE_2 _for a further 24 hrs.

### Intracellular detection of CD40L protein (CD154)

2 × 10^5 ^DCs were harvested post-transfection with CD40L mRNA and re-suspended in 250μL of Cytofix/Cytoperm solution (BD Biosciences) at 4°C. Cells were washed twice with 2 mL staining buffer (PBS, BSA, NaN_3_, and EDTA), re-suspended in 0.5 mL staining buffer and stored over night at 4°C. Cells were re-suspended in 2.0 mL Perm/Wash solution (BD Biosciences) for 15 minutes, centrifuged and re-suspended in 100μL Perm/Wash solution. 20μL of mouse anti-human CD40L PE and anti-human CD40 APC (BD Biosciences) or mouse IgG1 PE and IgG1 APC (BD Biosciences) was added to each DCs preparation collected and permeabilised at each time point, and incubated at 4°C for 30 minutes in the dark. Cells were washed twice with 1 mL Perm/Wash solution and re-suspended in staining buffer prior to flow cytometric analysis.

### Quantitation of IL-12 secretion from mature DC populations

Mature DCs generated as described above, were immediately cultured post-electroporation at a concentration of 1 × 10^6 ^cells/ml and supernatants collected after 24 hrs for the measurement of Il-12 or Il-10 secretion using BD Pharmingen IL-12 or IL-10 ELISA kits. Briefly, ELISA plates (BD Biosciences) were coated with anti-IL-12p70 or anti-IL-10 ELISA capture antibody in coating buffer for 24 hours at 4°C using BD Opt EIA reagent set B pH 9.5. Plates underwent blocking with 200μL per well 10%FCS/PBS for one hour prior to the addition of standards (BD Pharmingen) and supernatant samples, in duplicate, at 100μL per well and incubated at room temperature for 2 hrs. Plates were washed and anti-cytokine detection antibody added, incubated for one hour, the plates washed and solutions replaced with 100 μL of streptavidin-HRP and further incubated for one hour at room temperature. Again plates were washed and colour development substrates applied for 10–20 minutes, followed by cessation of colour development with stop solution. Plate analysis undertaken using Bio-Tek instruments ELx800 plate reader with KC junior software.

## Authors' contributions

IT conceived of the study, carried out its coordination, conducted experiments, drafted the manuscript and revised for important intellectual content. MA carried out molecular biology side of most experiments and analyzed the data, participated in the design of the study. XF created site-directed constructs as well as prepared the RNAs.

AH, JH and DC carried out the immunoassays. DH participated in the design and coordination of the study. CN contributed to the drafting of the manuscript and approved the final version to be published.
